# Independent Dental Journalism

**Published:** 1891-02

**Authors:** L. Ashley Faught

**Affiliations:** Philadelphia


### INDEPENDENT DENTAL JOURNALISM.[1]

BY L. ASHLEY FAUGHT, D.D.S., PHILADELPHIA.

Mr. President and Gentlemen,—I have no apology to offer to the profession to-night for inviting attention to the consideration of this subject. The very air for the last six months has been full of it; and while, perhaps, it has never been openly discussed on the floor of any association, it is nevertheless to my mind a topic upon which many, like myself, may desire information; and a comparison of ideas ought not to prove barren of beneficial results. Certainly the floor of an association like this is a fairer field of combat than the pages of any journal, dependent or independent; and the cool impartial opinions of readers and contributors

---

[1] Read at the meeting of the Odontological Society of Pennsylvania, November 1, 1890.

may weigh more justly in the balance than the mere statements of assertion and denial from persons in more interested positions.

There is no good reason why we should not freely discuss our journals, and I shall open the way for it. If we do not tell each other to-night our thoughts in reference to them, and what we regard as best in them, or needful for their improvement, the omission will not be due to lack of invitation or of opportunity.

In order that we may intelligently discuss the subject, a slight *résumé* may not be inappropriate, and to this end I propose the query, What is the object of dental journalism? The journals of any art, science, trade, or manufacture are primarily the outgrowth of a desire on the part of those most interested in such subjects, though widely separated in point of space, to communicate to each other, for mutual benefit, the progress made and the new ideas developed from day to day; and, secondarily, the result of a wish of their publishers to further their personal interests.

Dental journalism, therefore, found the springs of its birth in a desire to supplement the early meagre college training, and has ever since drawn its life from the veins of a profession to which it relates as a post-graduate course.

The little band of dental pioneers, shivering under the rude cold blast that usually meets early aspirations, turned from the closed doors shut by the powers then in control of the healing art, and sought to nurse the fitful flame of the smouldering spark by sweet communion of kindred hearts. The Baltimore *Dental Journal*, the *Dental Register*, the New York *Dental Recorder*, the *Dental Intelligencer*, and, best of all, the *Dental News Letter*, came into existence, and the dentists who wrote for them, though possessing the doctorate in medicine, signed their articles "John Brown, M.D., Dentist." Too small to properly represent the young nursling, the *Dental News Letter* was succeeded by the *Dental Cosmos*, and the imprint on its first page under the topic, "Our Enterprise," suggests that it seeks to "fairly cover the dentists' world of science and practice;" that "the meaning of the title is exactly the intention of the publishers," and that "the *Dental Cosmos* is pledged to the dental public to do whatever a journal can for the good cause of professional improvement, for the profession's advancement in its usefulness, self-respect, and public regard, and for strengthening fraternal courtesy, justice, and co-operation among the men who have the destiny and responsibility of the profession in their hands." This pledge was amply fulfilled during the succeeding years, and represented all that there was of any note in dental journalism up to 1872. At this date one whose memory is yet green, and whose professional spirit will ever be revered, in retiring from its editorship, stated that its primary object had ever been "the elevation of the professional standard to the highest possible point of excellence," . . . and "the views advanced have been offered for what they were worth, consulting more what I believed to be the best interests of the profession than what might prove to be popular, . . . and I desire that its record in the future, as an exponent of the interests and aspirations of the profession, should be equal if not superior to the past." With this benediction the *Dental Cosmos* passed from his hands, and in the same number of its issue the publisher states: "It is our intent and earnest desire to make the *Dental Cosmos* a practical exponent of the science and art of dentistry, . . . to supply all that the practical progress of the medical and dental professions can be made to afford." With the mantle of high professional spirit thus falling from the shoulders of one and resting so gracefully upon another, is it a matter of wonder that no true dentist to-day can afford to be without its pages, even though it may have fallen into a popular error?

From about the year 1872 up to the present time, as a result of natural professional progress, and perhaps stimulated from time to time by other minor impelling powers, the journalism of dentistry has greatly multiplied until the immediate furnishing seems most replete.

We sit not down to-day to a meagre table, but we have a choice of viands. It is perhaps inopportune, as well as difficult, to select and indicate any one dish as the most nutritious, but the seasoning in them all calls for notice and seems unpalatable in two respects.

As regards the pepper, most of the cooks seem to be in doubt which will suit best the popular palate,—red or black,—and so they use indiscriminately both,—"profession"—"specialty,"—and those who partake question whether the salt to be used shall be coarse or refined. Now, gentlemen, there are some among us who recognize that the real difficulty lies not in the salt at all, but with the pepper, which ought always to be red and never black. This can be best shown in a consideration of the first word of our title, "Independent Dental Journalism." The mixture of black and red pepper since 1883 became so evident that, with a view of sparing further infliction to sensitive nerves, I wrote a letter to the *Independent Practitioner*, under date of October 11, 1888, querying what profession could be possibly meant when one used that word again and again in articles on "Dentistry, a Specialty in Medicine?"

I may not be denied here a short quotation from my letter. "Every attentive reader of the dental and medical literature produced during the last five years will recognize the pertinent force of the above query. . . . From the time that Chapin A. Harris and his coadjutors made overtures to the medical colleges, endeavoring to have dental professorships incorporated in them, and the scornful rejection of their propositions, up to about 1883, dentistry was not confounded in thought with medicine by any one. It was understood to have its own literature, colleges, societies, etc.; and the medical fraternity lost no opportunity to give prominence to the fact that dentistry had nothing to do with medicine.[1] Dentistry as an independent profession, however, thrived and grew, and made for itself so prominent a position that its respectability and strength were felt far and wide. Medicine, ever jealous of her supposed prerogative, has noted the healthy pulse, and during the last five years has made great change in her attitude and from her original policy. During this period a persistent attempt has been made by the medical profession to have it understood that dentistry is a specialty in medicine.[2] The effort has filled our literature with much interesting reading bearing upon the subject, but has in nowise been able to make dentistry a specialty in medicine; but, rather, has continually emphasized the fact that the healing art is in the hands of two great distinct and separate branches,—medicine and dentistry. Each distinct and independent.

Every article that has been written, every speech that has been uttered, advocating dentistry as a specialty in medicine is filled with the expression, "the profession." Such emphasis shows how deeply implanted dentistry is as a "profession."

According to this view the salt has little to do with the taste, for the word "independent" does not concern a consideration of journalism separating from a supposed interest of a particular college, society, or manufacturing establishment (coarse salt), and conducted in the interest of the dental profession, by the profession, of the profession, and for the profession. (Refined salt.)

As another has tritely put it, "The question is not *where* is the literature of a profession purchased, but *what is its character?*" For my own part, gentlemen, if the manufacturer of dental requisites can produce a better journal, it matters not whether it emanates from a shop or a college, and God speed to him who makes journals

---

[1] See *Dental News Letter*, 1851, p. 109, and *Dental Cosmos*, 1874, p. 325.

[2] See *Dental Cosmos*, 1887, pp. 135, 405, 460, 516.

more useful to those who subscribe for and read them. Still declining to express an opinion as to which of the many is best, I venture the assertion, without fear of successful contradiction, that the house from which one of them emanates has done much to give to dentistry the cast of an independent profession. We may cut loose from the manufacturers so far as journalism is concerned, and we may by the most strenuous efforts endeavor to attach ourselves to the medical profession as a specialty; but we will never be successful in our identification with the latter, and we will always find ourselves classified with the former, as we now are by the census office, unless we seize upon the true meaning of "independent," and stand up for ourselves, and for what we are, as expressed by our title D.D.S.

Dr. Norman Kingsley voiced this in true old-fashioned Anglo-Saxon when he said, not long since, in speaking of a kindred topic, "Hardly a speaker has been on his feet that has not talked about 'our specialty' and in the next breath about 'our profession.'

"One gentleman, in his remarks about independent dental journalism, said, what I did not know until this evening, that this Society (New York Odontological) has committed itself unanimously to independent dental journalism. Now, as far as I can find out, the independent dental journalism which this Society has committed itself to, if it has at all, is made up of the veriest twaddle. Independent of what? Independent of a company of publishers that are able to publish a thoroughly creditable journal? Is that independent dental journalism? Not according to my mind at all. Independent dental journalism should represent dentistry in its independence.

"I tell you, gentlemen, that what is now doing more to menace the integrity of the dental profession than all else is not the men who are preparing materials and furnishing goods for dentists' use, but it is the class of men who are trying to attach dentistry to another profession."

This, gentlemen, is the truth,—sound solid truth,—and we ought as dentists to stand up for dentistry as dentistry, and, as an independent profession, engraving upon our cards and putting upon our printed matter, upon our window-sills and upon our door-plates, in fact upon all that goes before the public, not Dr. ——, but ——, D.D.S., thus acknowledging everywhere that we are dentists, and proud of being dentists; and start out plainly and boldly with real independent dental journalism.

